# Effect of hypothyroidism on the hypothalamic–pituitary–ovarian axis and reproductive function of pregnant rats

**DOI:** 10.1186/s12902-018-0258-y

**Published:** 2018-05-24

**Authors:** Jianran Sun, Cancan Hui, Tongjia Xia, Min Xu, Datong Deng, Faming Pan, Youmin Wang

**Affiliations:** 1 Department of Endocrinology, Institute of Endocrinology and Metabolism, The First Affiliated Hospital of Anhui Medical University, 218 Jixi Road, Hefei, 230022 Anhui China; 2 Department of Epidemiology and Biostatistics,School of Public Health, Anhui Medical University,81Meishan Road, Hefei, 230032 Anhui China

**Keywords:** Hypothyroidism, Hypothalamic–pituitary–ovarian axis, GnRH, GnRHR

## Abstract

**Background:**

This study aimed to detect changes in hormone levels in the hypothalamic–pituitary–ovarian axis in Sprague-Dawley (SD) rats with hypothyroidism, and identify differences in the pregnancy and abortion rates of female adult rats. The potential role of gonadotropin releasing hormone (GnRH) as the link between the hypothalamic–pituitary–ovarian axis and reproductive function regulated by thyroid hormones was also investigated.

**Methods:**

Female SD rats (*n* = 136) were causally classified into two groups: the normal-drinking-water group (*n* = 60) and the 0.05% propylthiouracil-drinking-water group (PTU 2 mg/kg/day, *n* = 76) to establish an adult rat model of hypothyroidism (6 weeks). Female and male rats at a ratio of 1:2 were used to establish a hypothyroidism pregnancy model. GnRH mRNA and GnRH receptor (GnRHR) expression in rats was detected using real time quantitative PCR(qRT-PCR) and immunohistochemistry, respectively.

**Results:**

The abortion rate differed significantly between the hypothyroidism pregnancy group and the normal pregnancy group (*P* < 0.05). No significant differences were found in the distribution of the GnRHR among the five nuclei (hypothalamic arcuate nucleus, hypothalamic ventromedial nucleus, hypothalamic anterior nucleus, paraventricular nucleus of the hypothalamus, and ventral premammillary nucleus) of the hypothalamus and ovary (*P* > 0.05). Hypothyroidism had no significant effect on GnRH mRNA expression in the hypothalamic–pituitary–ovarian axis in the four groups (normal control group, normal pregnancy group, hypothyroidism pregnancy group, and hypothyroidism group) (*P* > 0.05).

**Conclusions:**

Hypothyroidism had an adverse impact on pregnancy in rats and may affect the distribution of pituitary GnRHR, whereas it did not obviously affect the distribution of GnRHR in the nuclei of the hypothalamus and ovary. Hypothyroidism had no effect on GnRH mRNA expression.

**Electronic supplementary material:**

The online version of this article (10.1186/s12902-018-0258-y) contains supplementary material, which is available to authorized users.

## Background

The regulation of reproductive function is mainly accomplished by the hypothalamic–pituitary–ovarian axis. The amount of thyroid hormone (TH) contributes to maintaining the stability of the pituitary ovarian axis [1]. Metabolic disorders caused by abnormal thyroid function further increase the risk of infertility [2]. Gonadotropin releasing hormone (GnRH) plays an important role in the cascade mediating the release of reproductive hormones in the hypothalamic–pituitary–ovarian axis [3]. Clinical findings identified bidirectional communication between thyroxine and the hypothalamic–pituitary–ovarian axis; however, the underlying mechanism remains unclear [4]. In the present study, we sought to determine whether GnRH is the central link between the hypothalamic–pituitary–ovarian axis and reproductive function regulated by TH.

In primates, puberty is initiated by a surge of pulsatile GnRH that begins a prolonged phase of juvenile development in which the hypothalamic network regulates the release of GnRH, which is held in check by mechanisms that are poorly understood [5]. At the initial stage of infancy and prior to GnRH release, circulating gonadotropin levels are elevated and, in infantile female primates, are associated with blood estradiol levels in the adult range [6]. Therefore, primate adolescence is thought to be dominated by two primary postnatal switches [7]. During infancy, the first switch is activated and inhibits GnRH pulsation discharge, which leads to a hypogonadotropic condition that secures gonadal silencing before puberty. At the end of adolescent growth, the second switch is invoked, which leads to the recurrence of puberty in the discharge of pulsatile GnRH and the formation of the hypothalamic–pituitary–ovarian axis (gonadarche), the major physiological process underlying primate puberty [8].

The release of GnRH from approximately 1000 neurons within the hypothalamus in a pulsatile manner, which is characteristic of postnatal development in primates, is largely independent of the gonad [9]. Although the switch of pulsatile GnRH discharge leading to the recurrence of puberty has been researched widely, few studies have explored the causes of the inhibition of the GnRH pulse generators at the start of youth. In any case, the potential neurobiology is difficult to comprehend [10]. Under both conditions, one can assume that significant developmental variations in GnRH pulsation are associated with structural remolding of the GnRH pulse generator and/or with molecular variations in the hypothalamus.

Pulsed GnRH discharge during pubertal relapse depends on the allowable action of TH in this developmental phase. The action of TH during infancy is not related to the constraint that affects pulsatile GnRH release during infancy-youth switching or during pubertal reactivation of the pulse generators of GnRH [11]. TH deficiency during infancy also fails to have an effect on the timing of the pubertal resurgence of gonadotropin secretion [12].

## Methods

### Materials

Propylthiouracil (PTU) was purchased from Sigma-Aldrich (St. Louis, MO, USA). Distilled water with 3% H_2_O_2_ and mouse monoclonal antibody against human GnRH receptor (GnRHR) (clone F1G4) were purchased from Beijing ZhongShan Golden Bridge Biological Technology, Co., Ltd. (Beijing, China). Phosphate-buffered saline (2 mg/mL, pH: 7.4), bovine serum albumin, SYBR Premix Ex Taq II (Tli RNaseH Plus), ROX Reference Dye, and ROX Reference Dye II were purchased from Takara Bio Dalian Co., Ltd. (Dalian, China). Rat tri-iodothyronine (T_3_) enzyme-linked immunosorbent assay (ELISA) kit, rat thyroxine (T_4_) ELISA kit, and rat thyroid-stimulating hormone (TSH) ELISA kit were purchased from Shanghai Yuan Ye Biotechnology Co., Ltd. (Shanghai, China).

### Protocols and experimental design

All animal experiments were performed in accordance with the principles approved by the Animal Ethics Committee of Anhui Medical University. A total of 280 SD rats (7 days old, 55–70 g) were obtained from the Experimental Animal Center of Anhui Medical University. Food and tap water were continuously available in the cage top. Lights in the colony were on from 04:00 to 20:00 h and temperatures were maintained between 21 °C and 25 °C. All conditions and treatments were in accordance with the recommended humane conditions. The rats were randomly mated in polycarbonate cages (40 × 30 × 26 cm) with grid floors from 18:00 to 18:30 h each day, and the female-to-male sex ratio of the SD rats was 1:2. The period of mating was 3 days and the frequency was three times per day. Cages were checked daily, and the day in which an avaginal plug was secreted and detected was considered to be day 1 of gestation (G-1). Stainless steel wire lids and wood shavings were used as bedding material, and parturition occurred between G-21 and G-2. Pregnant rats were housed individually in polycarbonate cages (38 × 30 × 27 cm).

### Infertile female SD rats

The SD rats that were mated three times but did not secrete a vaginal plug were considered infertile.

### Establishment of a hypothyroid pregnant rat model

**Fig. 1:**
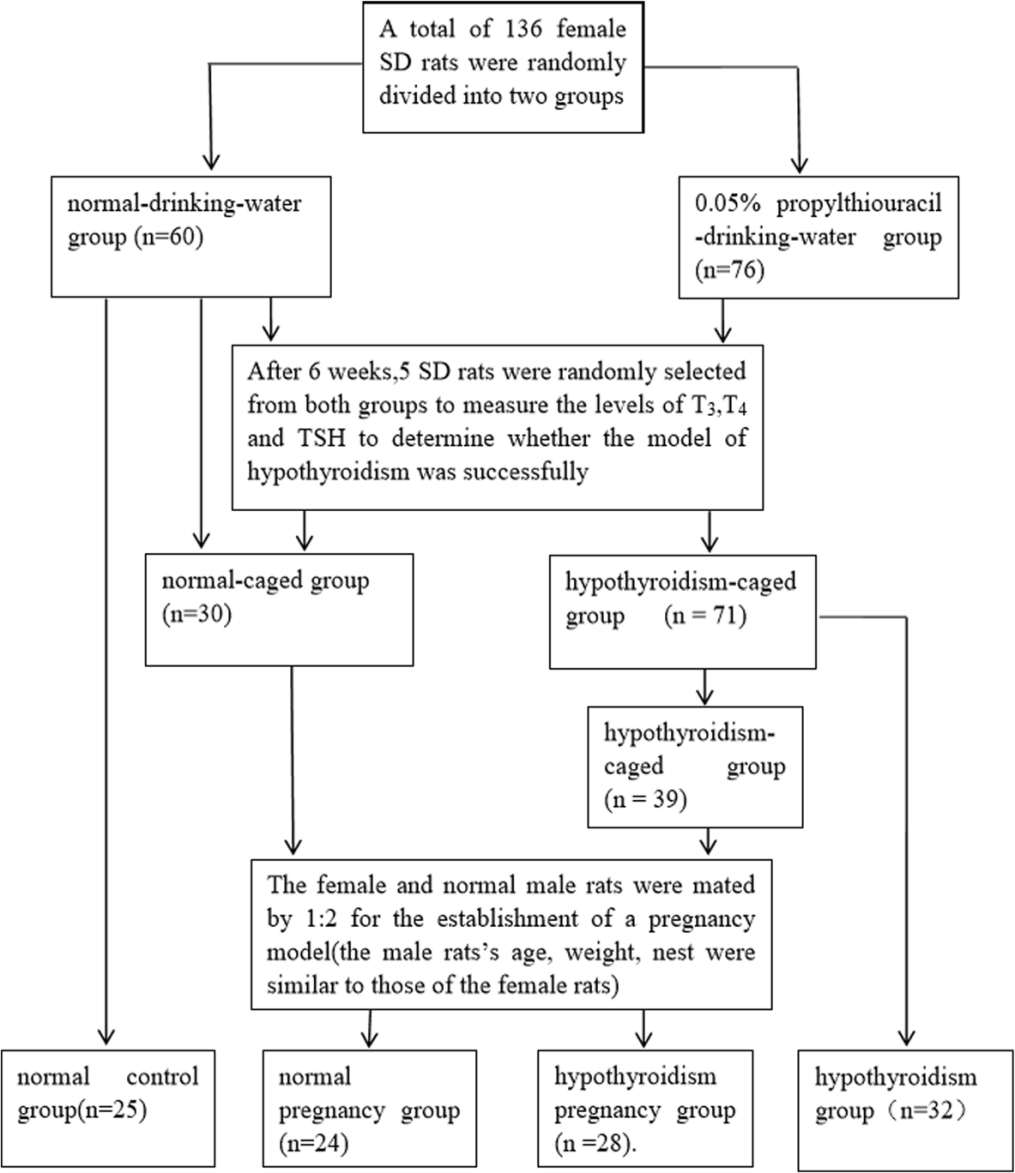
Establishment of the hypothyroid pregnant rat model

A total of 136 female SD rats were included and randomly divided into two groups, a normal-drinking-water group (*n* = 60) and a 0.05% PTU-drinking-water group (2 mg/kg/day, *n* = 76) to establish an adult rat model of hypothyroidism (6 weeks). Five rats were randomly selected from each group to measure the level of TH and determine whether the model of hypothyroidism was successfully established. Twenty-five SD rats from the normal-drinking-water group were randomly selected as the normal control group (*n* = 25). The remaining 30 SD rats in the normal-drinking-water group were subdivided into the normal caged-group (*n* = 30), and the remaining 71 rats in the PTU-drinking-water group were established as the hypothyroidism caged-group (*n* = 71). Thirty-two SD rats from the hypothyroidism caged-group were randomly picked as the hypothyroidism group (*n* = 32). The female and normal male rats at a respective ratio of 1:2 were mated to establish a pregnancy model, and the pregnant rats were further divided into the normal pregnancy group (*n* = 24) and the hypothyroidism pregnancy group (*n* = 28). One day after G-21, 11 rats that were not pregnant from the hypothyroidism pregnancy group and six rats that were not pregnant from the normal pregnancy group were humanely killed by cervical dislocation under anesthesia. Finally, there were 25, 24, 28, and 32 rats in the normal control group, normal pregnancy group, hypothyroidism pregnancy group, and hypothyroidism group, respectively. The establishment of the hypothyroid pregnant rat model is described in Fig. 1.

### Specimen preparation

After 12 h of fasting, the rats were anesthetized by intraperitoneal injection of 10% chloral hydrate (0.3 mL/100 g). Blood was collected from the abdominal aorta, and samples of brain tissues, pituitary tissues, and ovarian tissues were harvested. Portions of the samples were placed in 10% formaldehyde fixative, followed by gradient alcohol dehydration, xylene dewaxing, and serial sectioning.

### Immunohistochemistry staining detection

The tissue blocks were cut into 3–4 μm thick sections for histological analysis, dehydrated in an ascending series of ethanol, and dewaxed in xylene. For increased specificity and sensitivity, tissues were microwaved for 10 min for antigen retrieval, followed by cooling and rinsing in distilled water. Endogenous peroxidase activity was blocked with 3% H_2_O_2_ for 30 min at room temperature, followed by a serum-free protein block for 1 h at room temperature. Sections were incubated with antibodies against GnRHR (20 mg/mL) overnight at 4 °C. The streptavidin–peroxidase method was used to detect the antigen–antibody complexes, and diaminobenzidine was used as the chromogen substrate. The optical density values of stained tissues photographed with a Nikon Eclipse 80i fluorescence microscope (Tokyo, Japan) were quantified using Image Pro Plus 6.0 software (Maryland, USA).

### ELISA for determination of serum T_3_, T_4_, and TSH levels

Serum levels of T_3_, T_4_, and TSH were detected using ELISA following the kit protocol (Shanghai Yuanye Biological Technology Co., Ltd.). The activity of these hormones was used as a measure of thyroid function.

### qRT-PCR

RNA was isolated from tissues of the arcuate nucleus (ARC) of the hypothalamus of SD rats using the TRIzol reagent (Sigma–Aldrich, St Louis, MO, USA) according to the manufacturer’s instructions. mRNA levels were quantified using qRT-PCR. In brief, RNA preparations were used to synthesize cDNA in all qRT-PCRs, with a reaction volume of 20 μL, including primers (forward and reverse; details in Table 1), SYBR MIX, ddH_2_O, and 2 μL of RNA. The cycling conditions were as follows: denaturation for 0.5 min at 95 °C, followed by 40 cycles of amplification for 0.5 min at 60 °C and 5 s at 95 °C, and a final elongation step for 10 min at 72 °C. Every sample of cDNA (2 μL) was individually amplified using β-actin-specific primers to assess the RNA integrity and components of the reaction. qRT-PCR analyses were performed in duplicate with at least three individual RNA samples from each group during the period of development. The melting curves for every reaction were recorded to confirm the purity of the amplified product. The comparative cycle threshold (Ct) method was used to calculate the relative expression levels, and β-actin expression was used to normalize mRNA expression. The expression value of 7-day-old rat ARC was considered as the reference sample (Reference Value 1) for every period of development for comparisons with other groups. Every mean Ct value of β-actin was subtracted from the corresponding target-based Ct (GnRH) to obtain the ΔCt. The ΔCt of the 7-day-old rat was subtracted from each ΔCt of the period group to determine the ΔΔCt. Thus, the formula 2^−ΔΔCT^ was used to calculate the fold expression to compare the experimental groups with the 7-day-old rat samples. The relative quantity (RQ = 2^^(−△△Ct)^) value was the output by qRT-PCR, which is the comparison of the Ct value, transformed by mathematical formulas.

**Table 1:** Sequences of PCR primers

Gene	Primer	Sequence(5′^_^3’)	Accession no.
GnRH	GnRH-F	TCCAGCCAGCACTGGGTCCTA	NM_012767.2
	GnRH-R	GGGTTCTGCCATTTGATCCTC	
β-actin	β-actin-F β-actin-R	GGAGATTACTGCCCTGGCTCCTA GACTCATCGTACTCCTGCTTGCTG	NM_017008.4

*PCR* Polymerase chain reaction

### Statistical analysis

Statistical analysis was performed using IBM SPSS 16.0 (Armonk, NY, USA). The data of the normal distribution were represented as $\overline{x}\pm s$, and the data of the skewed distribution were represented as *M* (*P*_*25*_, *P*_*75*_). Differences between the two sets were compared using the Chi-square test for qualitative data. Differences between the two sets were compared by the paired t-test for normal distributions, and comparisons among groups were performed with one-way analysis of variance (one-way ANOVA). Differences between sets were also compared using the SNK-Q test [13, 14]. For non-normal distributions, differences between sets were compared with the Kruskal–Wallis test. Nemenyi test is a post hoc test that can be used after a Kruskal-Wallis test to indicate significant differences between the different groups. The *P*-value calibration method was used to compare the differences between the two sets with Fisher’s exact test using crosstabs of *n* < 40 and theoretical frequency [T ij = (n_i_ * m_j_)/n(n: total numbers of cases; n_i_: total numbers of the i row; m_j_: total numbers of the j column)] < 1. Differences with *P* < 0.05 were considered statistically significant.

## Results

### Establishment of a hypothyroid pregnant rat model

At the end of the 6-month period of drinking 0.05% PTU water, the female SD rats moved slowly. Their shins and hair became brownish yellow. Thyroid function was measured in blood samples collected from the angular vein. The results of paired t tests showed statistically significant differences in T_3_ (*t* = 13.749, *P* < 0.001), T_4_ (*t* = 9.644, *P* < 0.001, and TSH (*t* = 7.009, *P* < 0.001) between the normal-drinking-water group and the 0.05% PTU-drinking-water group. Serum T_3_ and T_4_ levels were lower and serum TSH levels were higher in the 0.05% PTU-drinking-water group than in the normal-drinking-water group (*P* < 0.05, both). The establishment of the hypothyroid rat model is described in Table 2.

**Table 2:** Establishment of hypothyroidism($\overline{x}\pm s$)

Items	normal-drinking- water group(*n* = 5)	0.05%PTU-drinking-water group(n = 5)	*t* value	*P* value
T_3_(ng/mL)	128.52 ± 6.57	66.27 ± 7.70^**^	13.749	< 0.001
T_4_(ug/dL)	232.76 ± 9.41	174.45 ± 9.67^**^	9.644	< 0.001
TSH(uIU/L)	1111.02 ± 84.83	1476.56 ± 79.75^**^	7.009	< 0.001

** *P <* 0.01,compared to normal-drinking- water group

### Comparison of the pregnancy and miscarriage rates of SD rats

There were significant differences in the conception rates between the normal pregnancy group and the hypothyroidism pregnancy group *(P* < 0.05) according to the results of the Chi-square test. The abortion rate differed significantly between the normal pregnancy group and the hypothyroidism pregnancy group as determined by Fisher’s exact test (*P* < 0.05). The results showed that the miscarriage rate increased and the pregnancy rate decreased in the hypothyroid pregnant SD rats. The pregnancy rate and miscarriage rate results are shown in Table 3.

**Table 3:** Pregnancy and miscarriage rates

Items	hypothyroidism pregnancy group (*n* = 39)	normal pregnancy group (*n* = 30)	χ^2^ value	*P* value
numbers of pregnancy	28	24		
numbers of miscarriage	10	0		
pregnancy rate(%)	71.4	80.0	0.002	0.096
miscarriage rate(%)	25.6^*^	0		0.036

In the cross table, n < 40 and *T* < 1; therefore, Fisher’s exact test is used (*P* = 0.036) and there is no χ^2^ value

### Immunohistochemical analysis of the expression of GnRHR in the hypothalamus, pituitary gland, and ovary in the four groups

**Fig. 2:**
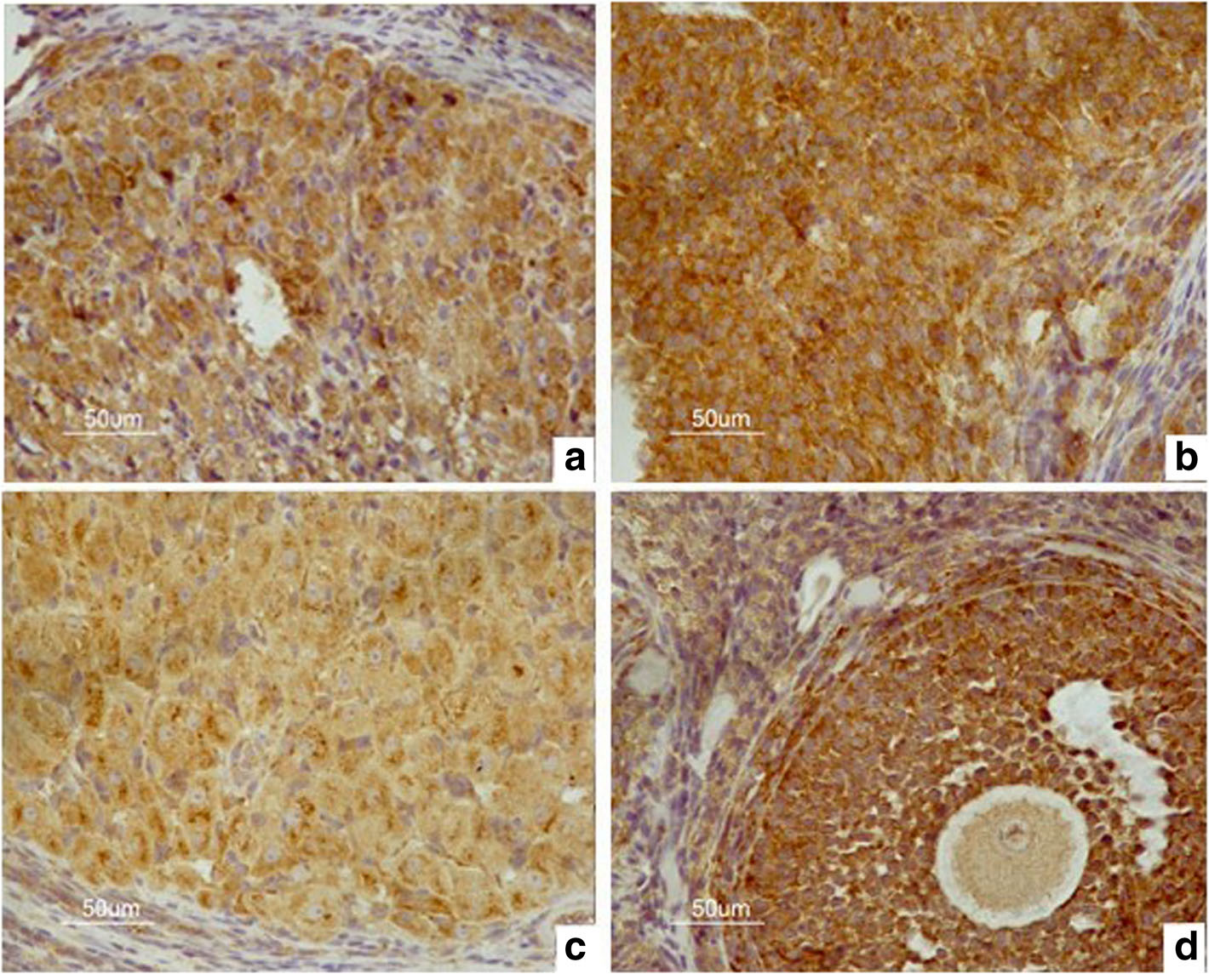
Immunohistochemical analysis of GnRHR expression in the hypothalamus in four groups. **a** hypothyroidism group. **b** normal control group. **c** normal pregnant group. **d** hypothyroidism pregnant group

**Fig. 3:**
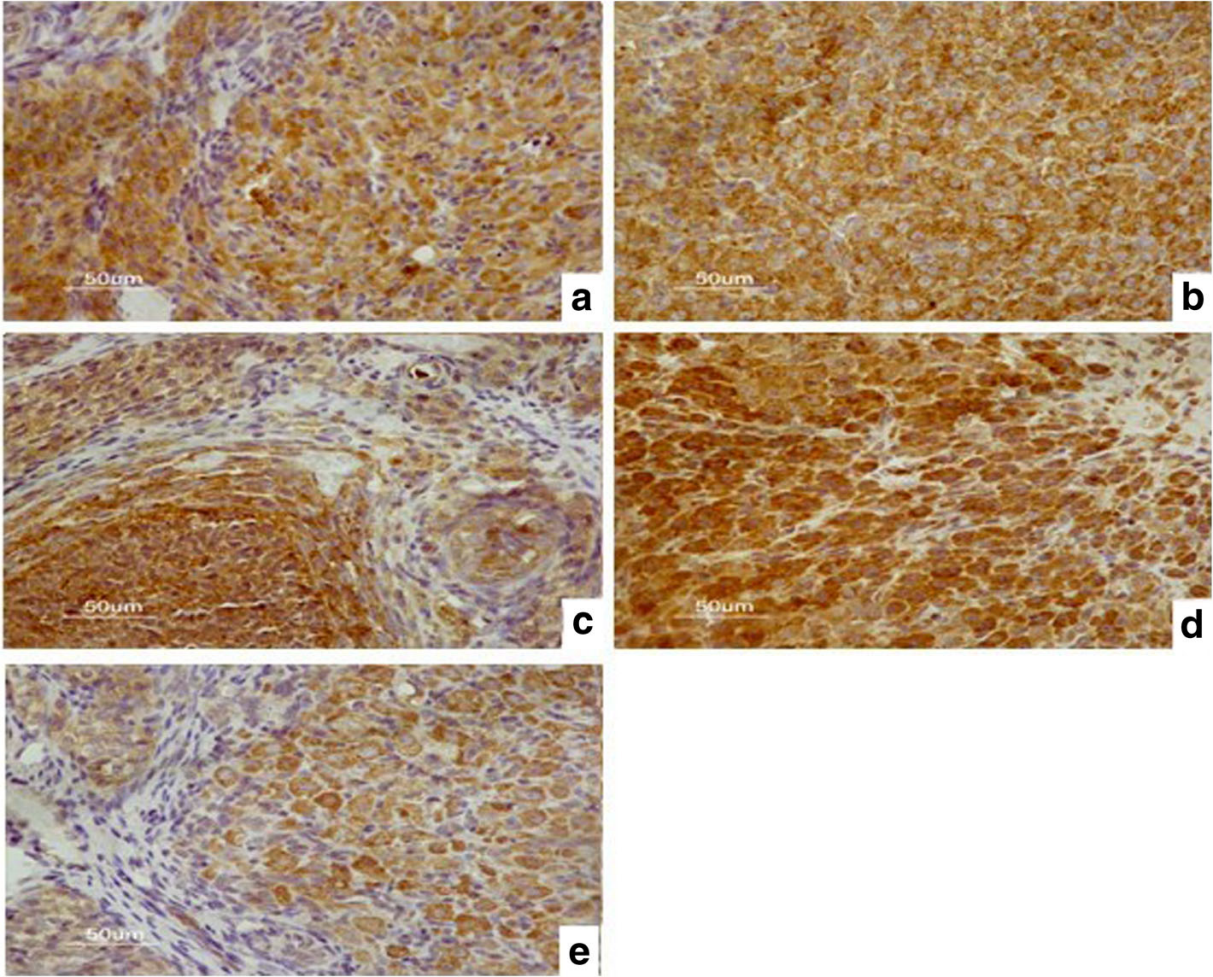
Hypothalamic nuclei. **a** hypothalamic arcuate nucleus. **b** hypothalamic ventromedial nucleus. **c** hypothalamic anterior nucleus. **d** paraventricular nucleus of hypothalamus. **e** ventral premammillary nucleus

**Fig. 4:**
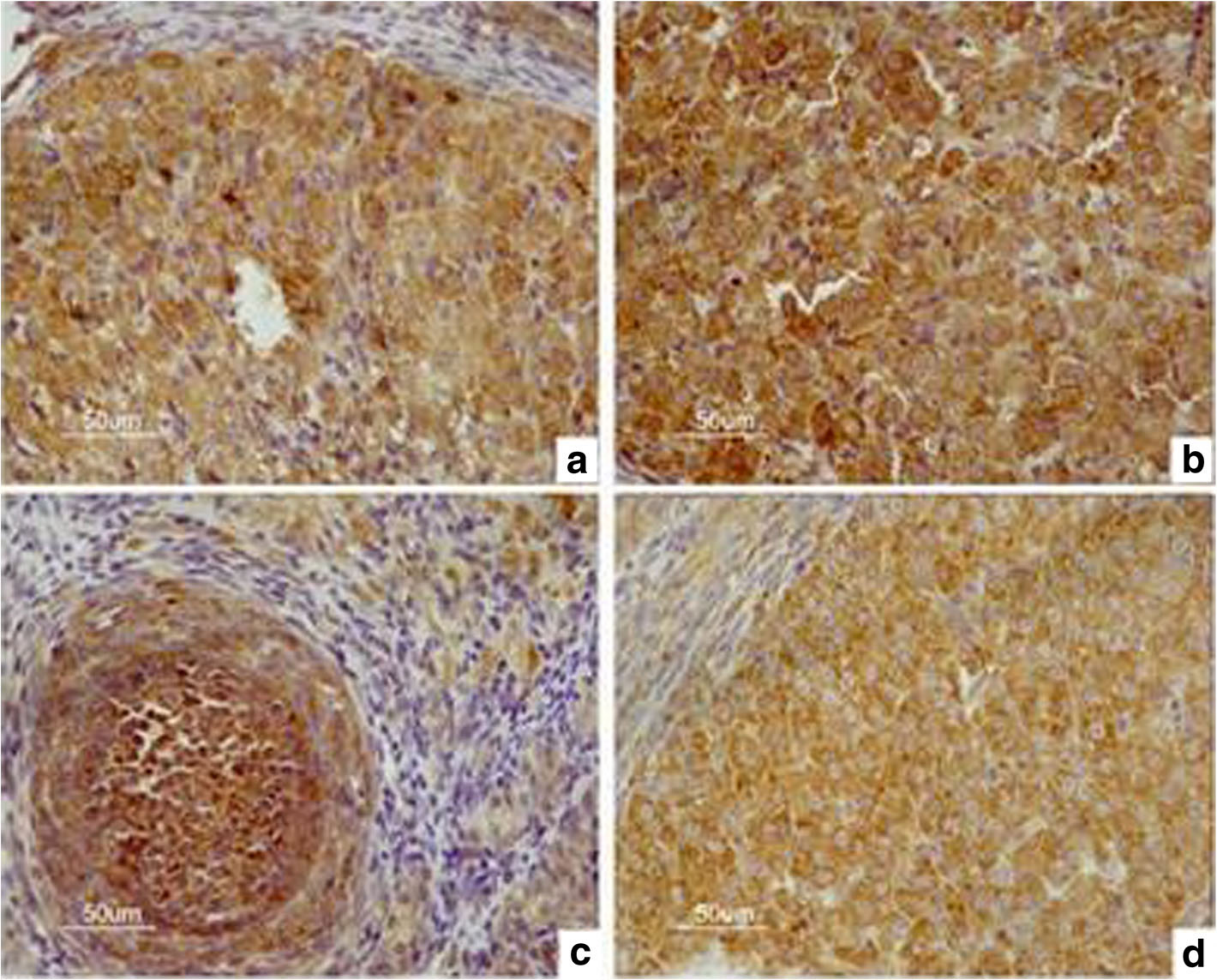
Immunohistochemical analysis of GnRHR expression in the pituitary in four groups. **a** hypothyroidism group**. b** normal control group. **c** normal pregnant group. **d** hypothyroidism pregnant group

**Fig. 5:**
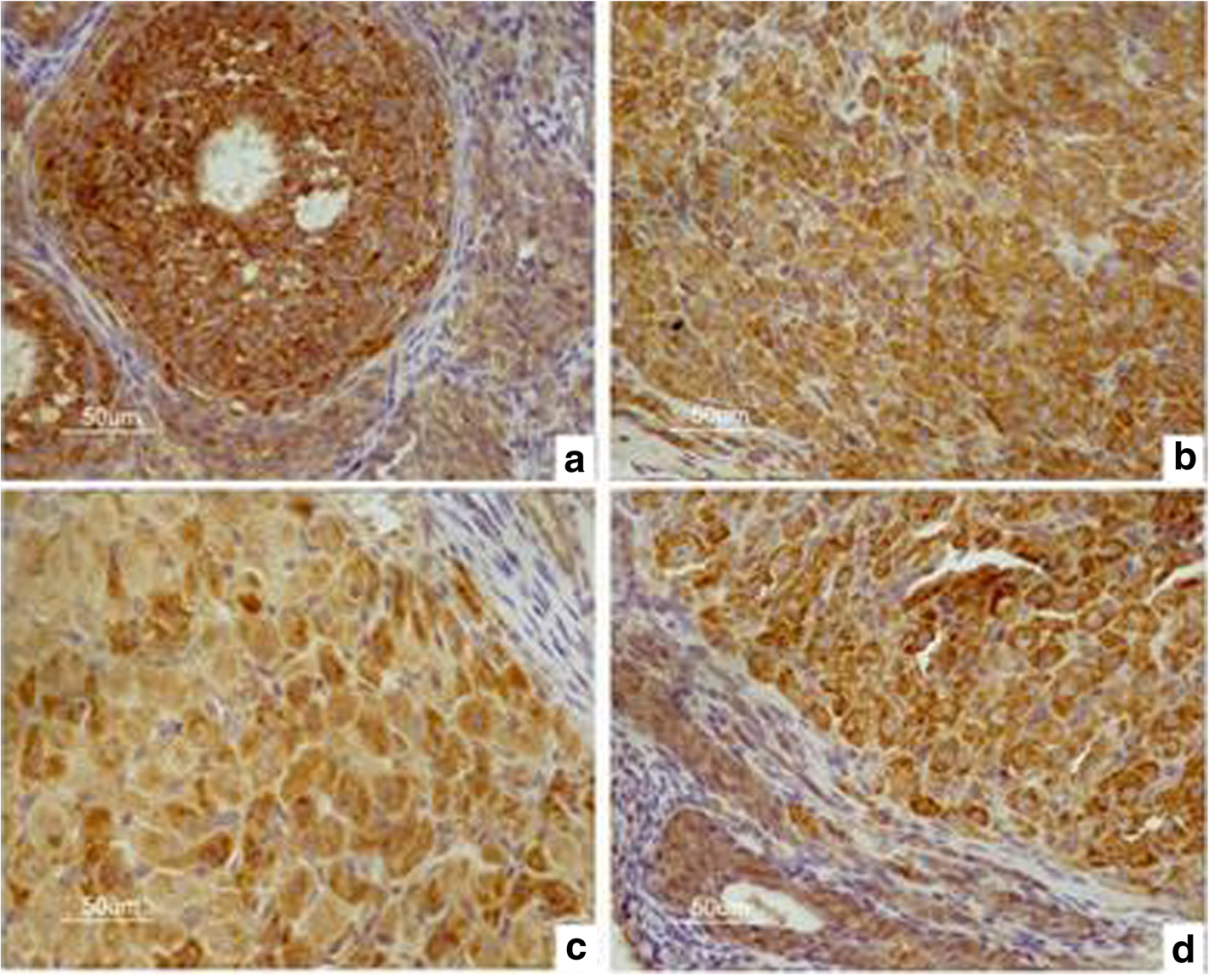
Immunohistochemical results of GnRHR expression in the ovary in four groups. **a** hypothyroidism group. **b** normal control group. **c** normal pregnant group. **d** hypothyroidism pregnant group

The immunohistochemistry results are shown in Figs. 2, 3, 4 and 5. The data of integral optical density values of pituitary GnRHR are consistent with skewed distribution. *M* (*P*_*25*_, *P*_*75*_) for figures used in the calculations are presented in a box plot. Similarly, the data of integral optical density values of ovarian GnRHR were consistent with normal distribution. $\overline{x}\pm s$ for figures used in the calculations are presented in a histogram. In the present study, we aimed to investigate the expression of GnRHR in the target gland, such as the pituitary gland, and ovary. Therefore, GnRHR distribution in the hypothalamus was used as a negative control. The histogram or box plot was added as a Supplementary file, without including the integral optical density values of the hypothalamus. The relative integral optical density values of pituitary and ovarian GnRHR in the four groups are shown in Additional file 1: Figure S1 and Additional file 2: Figure S2, respectively.

#### 3.1 The integral optical density values of hypothalamus GnRHR in the four groups are shown in Table 4

(1) In the normal control group, there were no statistically significant differences in the expression of GnRHR in the hypothalamus between the five nuclei by one-way ANOVA (*F* = 0.810, *P* = 0.531).

**Table 4:** Comparison of integral optical density values of hypothalamus GnRHR between four groups

Groups	hypothyroidism pregnancy group	hypothyroidism group	normal control group	normal pregnancy group	*F* value	*P* value
Numbers	28	32	25	24		
arc	(61.14 ± 42.84)	(46.47 ± 18.77)	(63.12 ± 42.14)	(48.87 ± 7.24)	0.373	0.774
vmh	(65.05 ± 36.71)	(48.31 ± 14.94)	(75.97 ± 29.75)	(70.22 ± 4.00)	1.346	0.291
ah	(64.94 ± 49.85)	(48.94 ± 5.84)	(73.75 ± 43.93)	(59.33 ± 23.37)	2.486	0.478
pa	(57.62 ± 30.70)	(42.85 ± 13.95)	(50.08 ± 17.37)	(67.07 ± 43.77)	0.591	0.670
pmv	(40.76 ± 17.26)	(46.22 ± 13.09)	(62.99 ± 32.10)	(46.95 ± 1.30)	0.517	0.676
*F* value	0.440	0.717	0.810	0.432		
*P* value	0.779	0.949	0.531	0.782		

arc:arcuate hypothalamic nucleus; vmh:ventromedial hypothalamic nucleus;

ah:anterior hypothalamic nucleus; pa:paraventricular nucleus of hypothalamus; pmv:ventral premammillary nucleus

(2) In the hypothyroidism group, there were no statistically significant differences between the five nuclei by one-way ANOVA (*F* = 0.717, *P* = 0.949).

(3) In the normal pregnancy group, there were no statistically significant differences between the five nuclei by one-way ANOVA (*F* = 0.432, *P* = 0.782).

(4) In the hypothyroidism pregnancy group, there were no statistically significant differences between the five nuclei by one-way ANOVA (*F* = 0.440, *P* = 0.779).

(5) There were no statistically significant differences in the hypothalamic arcuate (ar) nucleus between the four groups by one-way ANOVA (*F* = 0.373, *P* = 0.774).

(6) There were no statistically significant differences in the hypothalamic ventromedial (vmh) nucleus between the four groups by one-way ANOVA (*F* = 1.346, *P* = 0.291).

(7) There were no statistically significant differences in the hypothalamic anterior (ah) nucleus between the four groups by one-way ANOVA (*F* = 2.486, *P* = 0.478).

(8) There were no statistically significant differences in the paraventricular (pa) nucleus of hypothalamus between the four groups by one-way ANOVA (*F* = 0.591, *P* = 0.670).

(9) There were no statistically significant differences in the ventral premammillary (pmv) nucleus between the four groups by one-way ANOVA (*F* = 0.517, *P* = 0.676).

No differences in the distribution of GnRHR were identified between the five nuclei (ar, vmh, ah, pa, and pmv) of the hypothalamus.

#### 3.2 The integral optical density values of pituitary GnRHR in the four groups

(1) The Kruskal–Wallis test showed that there were statistically significant differences in the expression of the GnRHR in the pituitary between the four groups (*P* = 0.037).

(2) There were no statistically significant differences between the hypothyroidism pregnancy group and the hypothyroidism group according to the Nemenyi test (standard χ^2^ = 0.089, *P* = 0.929).

(3) There were no statistically significant differences between the hypothyroidism pregnancy group and normal control group (standard χ^2^ = 1.809, *P* = 0.071).

(4) Statistically significant differences were also found between the hypothyroidism pregnancy group and normal pregnancy group (standard χ^2^ = 2.345, *P* = 0.019).

(5) No statistically significant differences were observed between the hypothyroidism group and normal control group (standard χ^2^ = 1.720, *P* = 0.086).

(6) There were statistically significant differences between the hypothyroidism group and normal pregnancy group (standard χ^2^ = 2.268,*P* = 0.023).

(7) No statistically significant differences were detected between the normal control group and normal pregnancy group (standard χ^2^ = 0.779, *P* = 0.436).

To summarize, differences in the expression of the GnRHR in the pituitary were observed.

#### 3.3 The integral optical density values of ovarian GnRHR in the four groups

No statistically significant differences in the expression of the GnRHR in the ovary were observed between the four groups by one-way ANOVA (*F* = 0.544, *P* = 0.655).

## Effect of hypothyroidism on GnRH mRNA expression

**Fig. 6:**
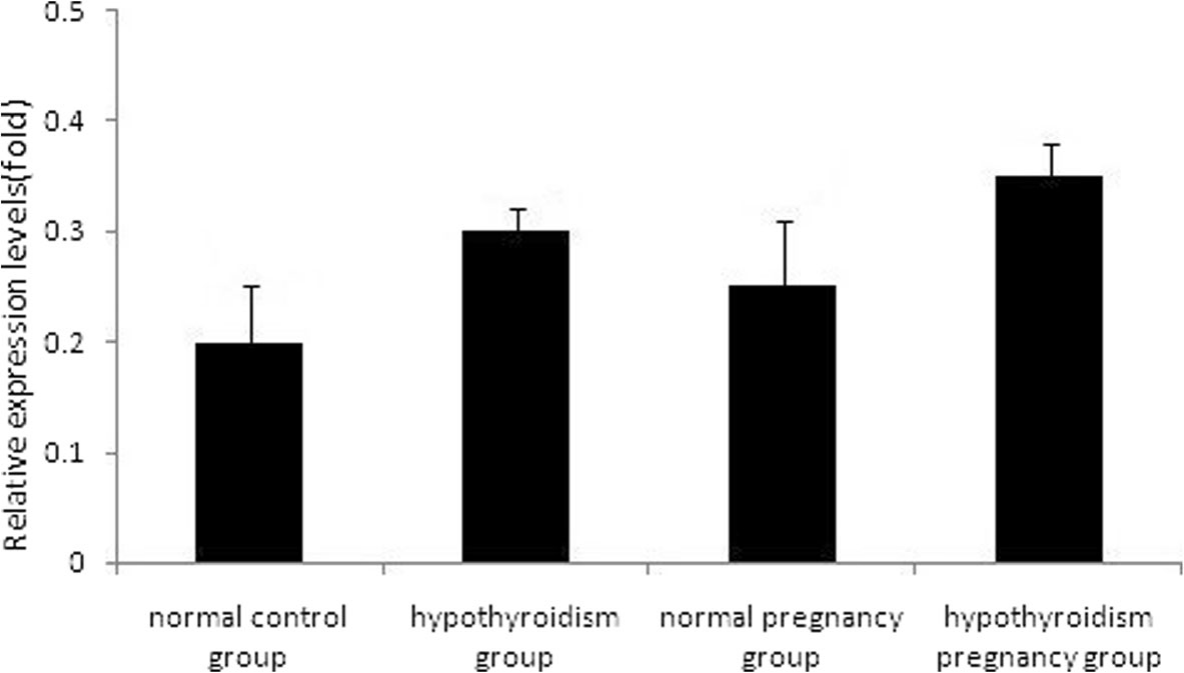
Relative mRNA levels of pituitary GnRH in the four groups determined using qRT-PCR, *P* > 0.05

**Fig. 7:**
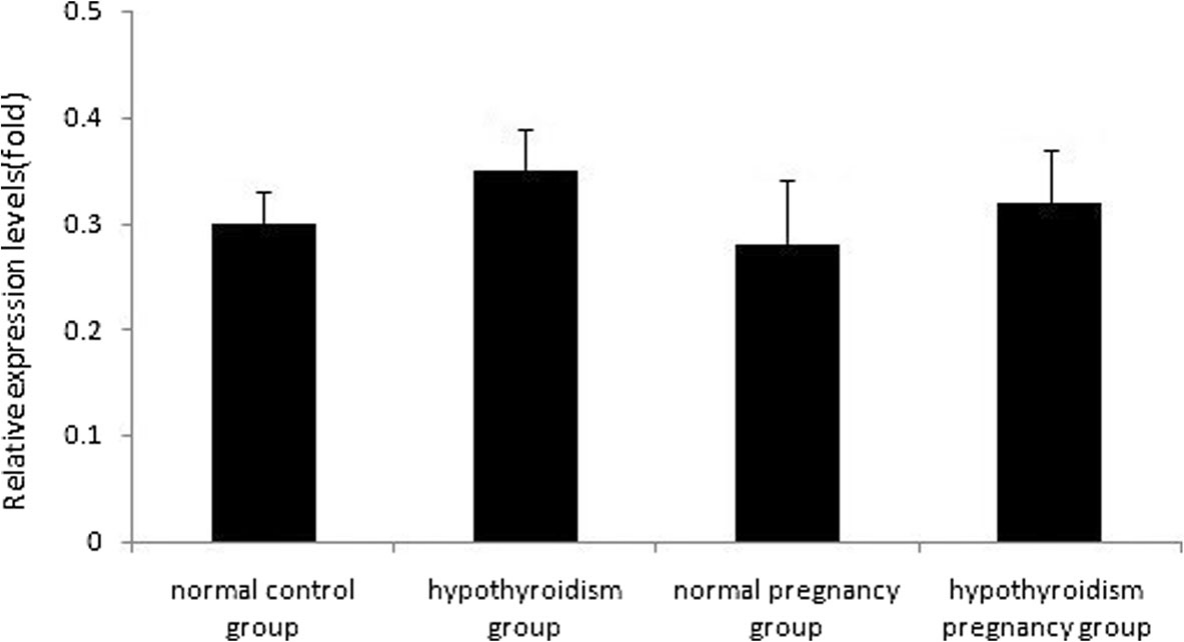
Relative mRNA levels of ovarian GnRH in the four groups determined using qRT-PCR, *P* > 0.05

The amplification curve of GnRH mRNA and β-actin mRNA showed an inverted S type. The amplification curves of GnRH mRNA ran parallel with those of β-actin mRNA, which indicated that qRT-PCR could detect β-actin in a broad range with high efficiency and in a short time. The solubility curve had a single peak, which indicated that primer specificity was high. This indicated that a single qRT-PCR product was used, and that the data obtained were reliable. The relative mRNA levels of pituitary and ovarian GnRH in the four groups are shown in Fig. 6 and Fig. 7, respectively.

### Comparison of the RQ values of hypothalamus GnRH mRNA between the four groups

There was no statistically significant difference by one-way ANOVA (*F* = 1.412, *P* = 0.296) (*P* > 0.05, details in Table 5).

**Table 5:** Comparison of RQ values of hypothalamus GnRH mRNA between four groups

Items	Hypothyroidism pregnancy group	Hypothyroidism group	normal control group	norma pregnancy group	*F* value	*P* value
C_T_	(21.42,29.96)	(21.96,23.83)	(20.02,24.27)	(21.56,24.69)	–	–
△C_T_	(5.72,15.29)	(5.60,8.30)	(3.90,7.19)	(6.45,10.00)	–	–
△△C_T_	(−4.27,5.29)	(−4.39,-1.70)	(−6.09,-2.81)	(−3.55,0.00)	–	–
RQ	(8.56 ± 9.85)	(16.79 ± 5.97)	(29.04 ± 27.71)	(7.62 ± 4.68)	1.412	0.296

### Comparison of the RQ values of pituitary GnRH mRNA between the four groups

There was no statistically significant difference by one-way ANOVA (*F* = 1.153, *P* = 0.368) (*P* > 0.05, details in Table 6).

**Table 6:** Comparison of RQ values of pituitary GnRH mRNA between four groups

Items	hypothyroidism pregnancy group	hypothyroidism group	normal control group	norma pregnancy group	*F*value	*P* value
C_T_	(30.03,31.98)	(31.79,33.24)	(31.56,32.93)	(32.60,32.73)	–	–
△C_T_	(11.82,12.18)	(11.76,33.24)	(12.53,13.32)	(13.35,14.19)	–	–
△△C_T_	(0.94,1.11)	(0.69,1.38)	(1.46,2.25)	(2.28,3.11)	–	–
RQ	(0.16 ± 0.07)	(0.78 ± 0.79)	(0.49 ± 0.31)	(0.42 ± 0.40)	1.153	0.368

### Comparison of the RQ values of ovarian GnRH mRNA between the four groups

There were no statistically significant differences by Kruskal–Wallis test (*χ*^*2*^ = 1.276, *P* = 0.077) (*P* > 0.05, Table 7).

**Table 7:** Comparison of RQ values of ovarian GnRHmRNA between four groups

Items	hypothyroidism pregnancy group	hypothyroidism group	normal control group	norma pregnancy group	χ^2^value	*P* value
C_T_	(31.72,31.93)	(31.46,34.40)	(30.94,35.30)	(31.51,32.09)	–	–
△C_T_	(8.82,9.81)	(5.71,7.86)	(3.39,10.17)	(9.46,10.24)	–	–
△△C_T_	(6.43,7.42)	(3.32,5.47)	(0.99,7.78)	(7.07,7.85)	–	–
RQ	(0.01,0.02)	(0.02,0.10)	(0.04,0.50)	(0.04,0.07)	1.276	0.077

## GnRH mRNA RQ values of the hypothalamus, pituitary gland, and ovary in the normal control group

There was a statistically significant difference between these groups (*χ*^*2*^ = 7.372, *P* = 0.025). The results of group-wise comparisons were as follows:

(1) There was no statistically significant difference in the value of GnRH mRNA RQ between the ovary and pituitary gland (*Z* = 0.889, *P* = 0.374)

(2) There was a statistically significant difference in the value of GnRH mRNA RQ between the ovary and hypothalamus (*Z* = 2.666, *P = 0.008*)

(3) There was no statistically significant difference in the value of GnRH mRNA RQ between the hypothalamus and pituitary (*Z* = 1.778, *P* = 0.075)

## Comparison of GnRH mRNA RQ values of the hypothalamus, pituitary gland, and ovary in the hypothyroidism group

There was a statistically significant difference between these groups (standard *χ*^*2*^ = 8.769, *P* = 0.012). Group-wise comparisons were as follows:

(1) There was no statistically significant difference in the value of GnRH mRNA RQ between the ovary and pituitary gland (*Z* = 1.177, *P* = 0.239)

(2) There was a statistically significant difference in the value of GnRH mRNA RQ between the ovary and hypothalamus (*Z* = 2.942, *P* = 0.003)

(3) There was no statistically significant difference in the value of GnRH mRNA RQ between the hypothalamus and the pituitary gland (*Z* = 1.765, *P =* 0.078)

## Comparison of GnRH mRNA RQ values of the hypothalamus, pituitary gland, and ovary in the normal pregnancy group

The difference was statistically significant (standard *χ*^*2*^ = 6.144, *P* = 0.046). Group-wise comparisons were as follows:

(1) There was a statistically significant difference in the value of GnRH mRNA RQ values between the ovary and pituitary gland (*Z* = 2.204, *P* = 0.028)

(2) There was a statistically significant difference in the value of GnRH mRNA RQ between the ovary and hypothalamus (*Z* = 2.205, *P* = 0.043)

(3) There was no statistically significant difference in the value of GnRH mRNA RQ between the hypothalamus and pituitary gland (*Z* = 0.329, *P* = 0.742)

## Comparison of GnRH mRNA RQ values of the hypothalamus, pituitary gland, and ovary in the hypothyroidism pregnancy group

The differences between these groups were statistically significant (standard *χ* ^*2*^ = 8.909, *P* = 0.012). Group-wise comparisons were as follows:

(1) There was no statistically significant difference in the value of GnRH mRNA RQ between the ovary and pituitary gland (*Z* = 1.706, *P* = 0.088)

(2) There was a statistically significant difference in the value of GnRH mRNA RQ between the ovary and hypothalamus (*Z* = 2.961, *P* = 0.003)

(3) There was no statistically significant difference in the value of GnRH mRNA RQ between the hypothalamus and pituitary gland (*Z* = 1.382, *P* = 0.167)

## Discussion

The mutual effects of the gonadal axis and the thyroid play a critical role in maintaining normal reproduction. Thyroid dysfunction can cause unnatural menstrual patterns, infertility problems, and ovulation disorders [15]. The high incidence of thyroid disease among women in sterile couples is well recorded in the literature [16].

In the present study, we demonstrated that hypothyroidism exerted an adverse effect on pregnancy in rats. Additionally, the results suggested that hypothyroidism in pregnant SD rats was associated with an increased miscarriage rate and a decreasing pregnancy rate. Hypothyroidism is associated with various conditions, such as placental abruption, pre-eclampsia, and intra-uterine fetal death [17]. Subclinical hypothyroidism is defined as a serum TSH level above the normal range despite normal serum free thyroxine levels. In addition, the side effects of subclinical hypothyroidism (SCH) include higher rates of premature delivery, abortion, and pregnancy-induced hypertension [18, 19]. Maraka et al. assessed (a) the effect of SCH during pregnancy on maternal and neonatal outcomes and (b) the impact of levothyroxine replacement therapy in these patients [20]. They found that compared with euthyroid pregnant women, pregnant women with SCH are at a higher risk of pregnancy loss [risk ratio (RR): 2.01, confidence interval (CI): 1.66–2.44], placental abruption (RR: 2.14, CI: 1.23–3.70), premature rupture of membranes (RR: 1.43, CI: 1.04–1.95), and neonatal death (RR: 2.58, CI: 1.41–4.73). The authors concluded that SCH during pregnancy is related to multiple adverse maternal and neonatal outcomes. The value of levothyroxine therapy in preventing these adverse outcomes remains uncertain.

The present results suggested that hypothyroidism affects the distribution of the pituitary GnRHR; however, we observed no evident effect on the distribution of the GnRHR of the ovary and hypothalamus. The GnRH neuronal system plays a regulatory role in the pituitary–gonadal axis hierarchy by regulating the secretion of pituitary luteinizing hormone and follicle-stimulating hormone. Variations of GnRH neurons are critical for regulating pubertas and the oestrous cycle. It is noteworthy that a “GnRH surge” triggers ovulation, and GnRH pulse secretion is not under seasonal control [21].

Although studies clearly show how hormones and other factors affect propagation, the brain remains a “black box” in that model. GnRH neurons serve as the ultimate pathway for controlling propagation; however, identification of the GnRH neurons involved in pulsatile secretion and how steroids and other factors modulate their activity remain unresolved issues, as indicated by the latest surveys among researchers. In the next sections, some key tests are reviewed. Because thyroid hormones (THs) are critical mediators, new evidence supporting this role is needed as well as evidence showing that THs directly affect GnRH neurons and may affect their developmental destiny and functionality in the brains of adults [22].

Unlike other neuroendocrine systems, GnRH neurons are scattered in various forebrain areas from the olfactory bulb to the hypothalamus, rather than in discrete nuclei or areas [23]. Since several GnRH cells form synaptic connections with other neurons and are distributed widely, it makes sense to question whether any GnRH neurons are neuroendocrine area-specific, that is, projected to median uplift, the location of release of the peptide into the pituitary portal system, which may indicate the export of neurons to different remote regions [24]. In the classic theory, GnRH itself is released from hypothalamic nuclei. However, three different forms of GnRH have been reported: hypothalamic GnRH or GnRH-I, mid brain GnRH or GnRH-II, and GnRH-III, which are present in various species of protochordates and vertebrates [25]. Although the hypothalamus and pituitary are the principal sources and target sites for GnRH, several recent reports suggested the presence of extra-hypothalamic GnRH and GnRH receptors in various reproductive tissues such as ovaries, placenta, endometrium, oviducts, testes, prostate, and mammary glands [26, 27]. GnRH in non-hypothalamic reproductive tissues may have interfered with our experimental results; therefore, we were unable to determine the effect of hypothyroidism on GnRH mRNA expression.

Lehman [28] and others who studied ewes as models performed most of the work in this area. Thyroidectomized ewes are different from intact animals because they do not exhibit a reduction in GnRH secretion in the pituitary portal vein. Nevertheless, thyroxine substitution reverses this effect and converts thyroid-removed ewes from anestrus to the nursery stage. The underlying participation of the thyroid is interesting, because THs are necessary for the standard mature morphology of the central nervous system.

THs act through particular receptors (THRs) that belong to the transcriptional active nuclear receptor superfamily [29]. Two THR isoforms (α and β) have been identified, and each has known subtypes (α1, α2 and β1, β2). Although THRs are located in various tissues, in the brain, there are fewer phenotypic records of specific neuronal populations that bind to THs. Surprisingly, GnRH secretion and GnRHR mRNA levels seem to be affected by the thyroid state [30]. The hypothesis that GnRH neurons include THRs has been partially sustained. In a study of double-tagging immunocytochemistry, Lehman et al. demonstrated the existence of a THR in models of GnRH neurons in hamsters (28%) and sheep (46%) [28]. Preliminary results indicate that a β2 isoform exists in GnRH neurons of sheep. It is interesting that GnRH neurons involve nuclear THR, and that nuclear THR seems to exist in the relevant glial cell nuclei. These findings indicate that THs have a direct effect on the level of GnRH neurons. A possible interpretation is that THs affect GnRH neurons by facilitating gene expression, which is required for the pulsatile release of GnRH [31].

We hypothesized that GnRH neurons may be susceptible to TH development because they express THRs [32]. To test this, rat pups were induced to develop hypothyroidism through feeding (0.4% PTU water) and drinking of 0.1% PTU water from birth until 25 days of age. At 25 days, the pups were ablactated and no further PTU therapy was performed. At 150 days, the animals were immolated, and paired tests were performed to record body weight and brain weight. In addition, the brain was immunocytochemically stained for GnRH. In the hypothyroid group, the quantity of GnRH neurons was almost double that of the saline treated control in the anterior and lateral hypothalamus. There were no differences in other areas (tendon, medial septum/diagonal band, anterior visual area, and medial basal hypothalamus) between the hypothyroid and the control pups. It is conceivable that the increase in GnRH neurons was due to the disturbance of the standard development-associated GnRH cell loss or loss of phenotype in those regions [28]. Therefore, THs may affect GnRH system development because of their presence in other brain areas [32]. Our studies in SD rats indicated that hypothyroidism may affect the allocation of the pituitary GnRHR; however, whether THs play a role in the ovaries and hypothalamus and the nature of their involvement remains unclear, and there are few relevant reports in the literature.

## Conclusions

The present study showed that the GnRHR is expressed in tissues of the hypothalamic–pituitary–ovarian axis in pregnant SD rats. The expression of the GnRHR in the pituitary gland is related to the GnRH-induced synthesis and release of gonadotropins. The presence of the GnRHR in the ovary points to either direct effects of hypothalamic GnRH on ovarian function or paracrine/autocrine effects of ovarian GnRH. Because THs may act as potential mediators of GnRH neurons, hypothyroidism may affect the distribution of the pituitary GnRHR [21]. A limitation of this study was the inability to detect the frequency and amplitude of GnRH release. We were therefore unable to determine whether hypothyroidism affects the distribution of GnRHR in the nuclei of the ovary. The negative results may indicate the lack of sensitivity of the staining for measuring GnRHR expression. In addition, immunohistochemistry is only semi-quantitative at best.

Recent research in SD rats yielded new insights into which GnRH neurons mediate pulsatile secretion, the circuitry by which steroids may regulate reproductive transitions, and the potential for plasticity in that circuitry [33]. A number of important questions remain to be answered. From a long-term perspective, elucidating the mechanisms underlying the effect of THs on GnRH secretion may provide the basis for the development of new approaches to regulate fertility in animals and humans [34].

## Additional files

Additional file 1: **Figure S1.** Relative integral optical density values of pituitary GnRHR in the four groups. ^*^P < 0.05, compared with normal pregnancy group; ^#^*P* < 0.05, compared with normal pregnancy group. (TIF 1863 kb)

Additional file 2: **Figure S2.** Relative integral optical density values of ovarian GnRHR in the four groups. (TIF 2805 kb)

## Abbreviations

Ah nucleus: hypothalamic anterior nucleus
Ar nucleus: hypothalamic arcuate nucleus
ELISA: enzyme-linked immunosorbent assay
FT_4_: free thyroxine
GnRH: gonadotropin releasing hormone
GnRHR: GnRH receptor
Pa nucleus: paraventricular nucleus of hypothalamus
Pmv nucleus: ventral premammillary nucleus
PTU: propylthiouracil
qRT-PCR: real time quantitative PCR
SD rats: Sprague-Dawley rats
T_3_: tri-iodothyronine
T_4_: thyroxine
TH: thyroid hormone
THRs: thyroid hormone receptors
THs: thyroid hormones
TSH: thyroid-stimulating hormone
Vmh nucleus: hypothalamic ventromedial nucleus
