# Supplementary figures and images for: Effect of hypothyroidism on the hypothalamic–pituitary–ovarian axis and reproductive function of pregnant rats
Source: BMC Endocr Disord. 2018 May 24;18:30. doi: 10.1186/s12902-018-0258-y (PMC5968710; doi:10.1186/s12902-018-0258-y)

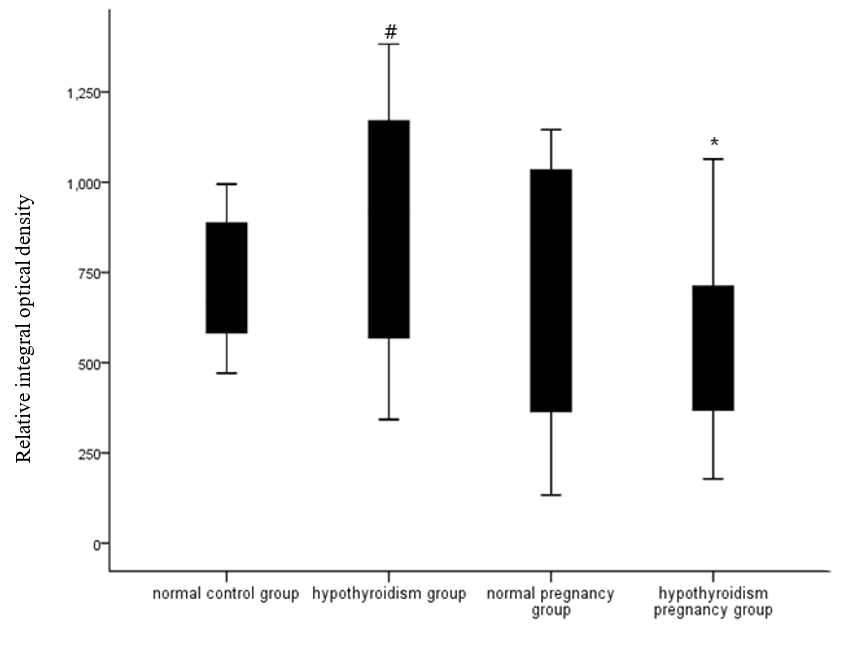

Supplement: Supplementary file 1 — Figure S1. Relative integral optical density values of pituitary GnRHR in the four groups. *P < 0.05, compared with normal pregnancy group; #P < 0.05, compared with normal pregnancy group. (TIF 1863 kb) [file 12902_2018_258_MOESM1_ESM.tif]

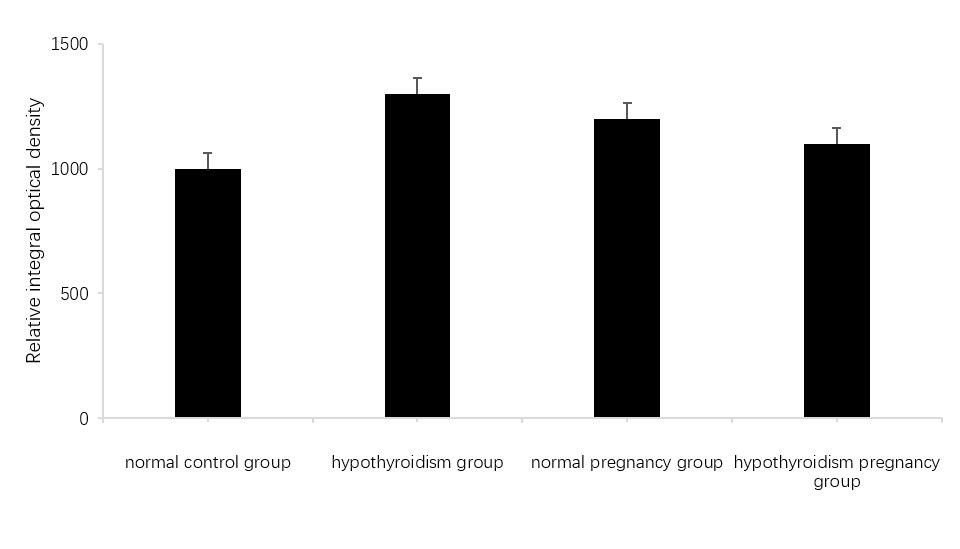

Supplement: Supplementary file 2 — Figure S2. Relative integral optical density values of ovarian GnRHR in the four groups. (TIF 2805 kb) [file 12902_2018_258_MOESM2_ESM.tif]
